# Deep Learning and Device-Assisted Enteroscopy: Automatic Detection of Gastrointestinal Angioectasia

**DOI:** 10.3390/medicina57121378

**Published:** 2021-12-18

**Authors:** Miguel Mascarenhas Saraiva, Tiago Ribeiro, João Afonso, Patrícia Andrade, Pedro Cardoso, João Ferreira, Hélder Cardoso, Guilherme Macedo

**Affiliations:** 1 Department of Gastroenterology, São João University Hospital, Alameda Professor Hernâni Monteiro, 4200-427 Porto, Portugal; tiagofcribeiro@outlook.com (T.R.); joaoafonso28@gmail.com (J.A.); anapatriciarandrade@gmail.com (P.A.); pedromarilio@gmail.com (P.C.); hc@sapo.pt (H.C.); guilhermemacedo59@gmail.com (G.M.); 2 WGO Gastroenterology and Hepatology Training Center, 4200-427 Porto, Portugal; 3 Faculty of Medicine of the University of Porto, Alameda Professor Hernâni Monteiro, 4200-427 Porto, Portugal; 4 Department of Mechanical Engineering, Faculty of Engineering of the University of Porto, Rua Dr. Roberto Frias, 4200-465 Porto, Portugal; j.ferreira@fe.up.pt

**Keywords:** device-assisted enteroscopy, angioectasia, gastrointestinal bleeding, artificial intelligence, convolutional neural networks, deep learning

## Abstract

*Background and Objectives*: Device-assisted enteroscopy (DAE) allows deep exploration of the small bowel and combines diagnostic and therapeutic capacities. Suspected mid-gastrointestinal bleeding is the most frequent indication for DAE, and vascular lesions, particularly angioectasia, are the most common etiology. Nevertheless, the diagnostic yield of DAE for the detection of these lesions is suboptimal. Deep learning algorithms have shown great potential for automatic detection of lesions in endoscopy. We aimed to develop an artificial intelligence (AI) model for the automatic detection of angioectasia DAE images. *Materials and Methods:* A convolutional neural network (CNN) was developed using DAE images. Each frame was labeled as normal/mucosa or angioectasia. The image dataset was split for the constitution of training and validation datasets. The latter was used for assessing the performance of the CNN. *Results*: A total of 72 DAE exams were included, and 6740 images were extracted (5345 of normal mucosa and 1395 of angioectasia). The model had a sensitivity of 88.5%, a specificity of 97.1% and an AUC of 0.988. The image processing speed was 6.4 ms/frame. *Conclusions*: The application of AI to DAE may have a significant impact on the management of patients with suspected mid-gastrointestinal bleeding.

## 1. Introduction

Obscure gastrointestinal bleeding (OGIB) comprises gastrointestinal bleeding without evident etiology after the performance of conventional esophagogastroduodenoscopy (EGD) and colonoscopy. It accounts for approximately 5% of all gastrointestinal hemorrhages and represents the most common indication for the performance of device-assisted enteroscopy (DAE) [1,2]. Most often, the source of bleeding is located in the small bowel, particularly in areas beyond the reach of conventional EGD and colonoscopy (i.e., between the second duodenal portion and distal ileum). Recent advances in imaging and endoscopic tools for the investigation of the small bowel led to a shift in definitions from OGIB to mid-gastrointestinal bleeding or small bowel bleeding [3], reserving OGIB for situations in which the bleeding source was not identified after assessment of the full length of the gastrointestinal tract [4].

Enteroscopy, and particularly DAE (i.e., single or double-balloon enteroscopy and spiral enteroscopy), plays a significant role both in the diagnosis and in the treatment of patients presenting with OGIB. A meta-analysis estimated a diagnostic yield of approximately 56% for double-balloon enteroscopy (DBE) in the setting of small bowel bleeding [5]. This modest diagnostic yield increases to 75% if DBE is performed after a first exploratory capsule endoscopy (CE) [5]. The modest diagnostic yields of DBE may explain the significative number of inconclusive DAE exams after a positive CE. Small bowel angioectasia is the most common bleeding source detected in the investigation for small bowel bleeding, accounting for up to 30% of cases [6]. However, the diagnostic yield of DAE, and particularly of DBE, is as low as 24% [7]. Moreover, in addition to enabling access to the small bowel, DAE detects either upper gastrointestinal lesions or colonic lesions in approximately 25% of patients presenting with OGIB [8]. 

The application of artificial intelligence (AI) algorithms for the automatic analysis of endoscopic images has been the focus of intensive research. Indeed, several systems were developed for implementation in EGD, colonoscopy and CE [9,10,11]. Deep learning systems, and particularly convolutional neural networks, allow the analysis of large image datasets with high performance. However, to date, no AI systems have been developed for the application to DAE. Additionally, no deep learning algorithms have been developed for the automatic detection of angiectasia in different segments of the gastrointestinal tract. This study aims to build and develop an AI system based on a convolutional neural network (CNN) for the automatic detection of angiectasia using DAE images.

## 2. Materials and Methods

### 2.1. Study Design

All patients undergoing DAE (either single-balloon enteroscopy or DBE) between January 2020 and May 2021 at a single center (São João University Hospital, Porto, Portugal) were enrolled in this study (*n* = 72). All procedures were recorded as a video file. Each full-length video was reviewed, and images retrieved from these examinations were used for the development, training and validation of a CNN algorithm for automatic detection of angioectasia. These images comprised still frames extracted by decomposition of each video. The segmentation of each video into frames was performed using a dedicated video software (VLC media player, Paris, France). Each frame was assessed by two endoscopists with expertise in DAE (PA and HC). The final classification of any frame (as normal or as showing angioectasia) required a consensus between both experts. When consensus could not be reached, the frame was excluded. Finally, 6740 images were included. 

This study was approved by the ethics committee of São João University Hospital/Faculty of Medicine of the University of Porto (No. CE 188/2021) and was conducted with respect to the declaration of Helsinki.

### 2.2. Device-Assisted Enteroscopy Procedure, Data Collection and Definition

All DAE (either single-balloon enteroscopy or DBE) were performed by two experienced endoscopists with expertise in DAE, each with >200 DAE procedures prior to this study. The procedures were performed with two different enteroscope models: DBE with Fujifilm EN-580T and single-balloon enteroscopy Olympus EVIS EXERA II SIF-Q180). Each system was assisted by an overtube. Both antegrade and retrograde DAE procedures were included. Images from the stomach, small bowel and colon were retrieved. Each extracted frame was analyzed for the presence of angioectasia. Angioectasia was defined as a well-demarcated bright red lesion consisting of tortuous and clustered capillary dilatations within the mucosal layer.

### 2.3. Development of the CNN

**Figure 1:**
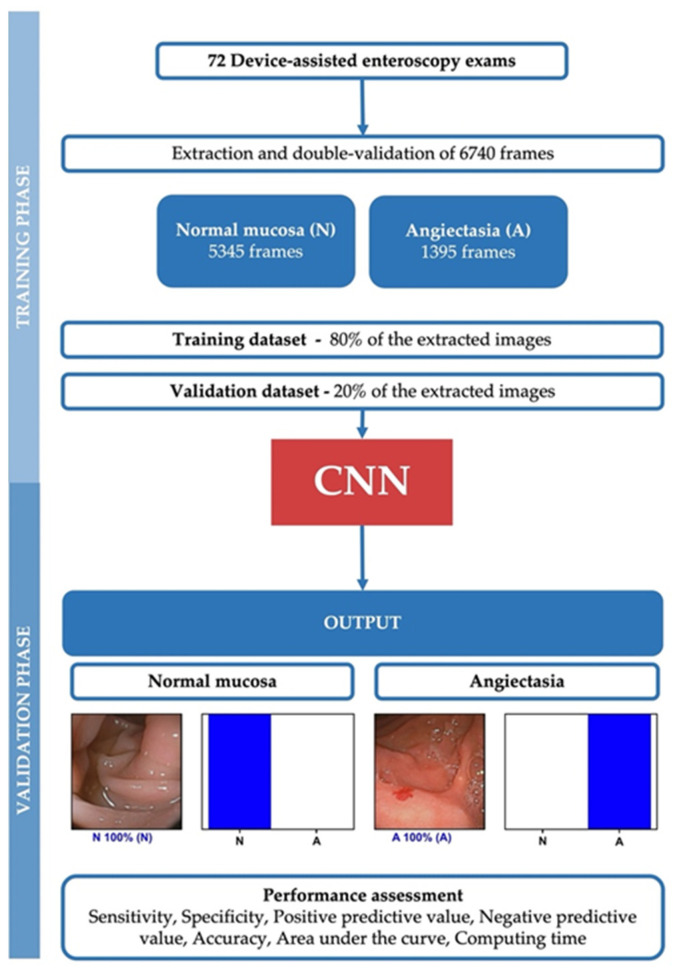
Study flowchart for the training and validation phases. N—Normal mucosa; A—Angioectasia.

A deep learning CNN was developed with the aim to automatically identify and differentiate angioectasia from normal mucosa on DAE images. From the collected pool of images (*n* = 6470), 1395 contained angioectasia, and the remaining showed normal mucosa. This pool of images was split for the constitution of training and validation datasets. The training dataset was composed of 80% of the extracted images (*n* = 5392). The remaining 20% was used as the validation dataset (*n* = 1348). The validation dataset was used for assessing the performance of the CNN. A flowchart summarizing the study is presented in Figure 1.

The Xception model was used to create the CNN, and its weights were trained on ImageNet. The convolutional layers of the model were kept to transfer this learning to our data. We removed the last fully connected layers and attached fully connected layers based on the number of classes we used to classify our endoscopic images. Two blocks, each with a fully connected layer followed by a dropout layer of 0.3 drop rate, were used. Following these two blocks, we added a dense layer with a size defined as the number of categories (three) to classify. The learning rate of 0.0001, batch size of 16, and the number of epochs of 100 were set by trial and error. We used Tensorflow 2.3 and Keras libraries to prepare the data and run the model. The analyses were performed with a computer equipped with a 2.1 GHz Intel^®^ Xeon^®^ Gold 6130 processor (Intel, Santa Clara, CA, USA) and a double NVIDIA Quadro^®^ RTX™ 4000 graphic processing unit (NVIDIA Corporate, Santa Clara, CA, USA).

### 2.4. Model Performance and Statistical Analysis

**Figure 2:**
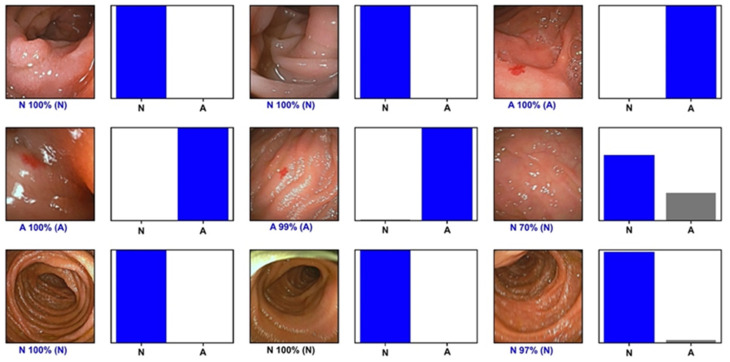
Output provided by the network. Each bar represents the probability calculated by the algorithm. The finding with the highest probability was outputted as the predicted classification. The blue bar represents a correct prediction. N—normal mucosa; A—Angioectasia.

For each image, the trained CNN calculated the probability for each category (angioectasia vs. normal mucosa). A higher probability represented greater confidence in the CNN’s prediction. The category with the highest probability score was output as the CNN’s predicted classification (Figure 2). The primary outcome measures included sensitivity, specificity, positive and negative predictive values, and the accuracy in differentiating between images showing angioectasia and images with normal mucosa. Moreover, we used receiver operating characteristic curve analysis and area under the ROC curve (AUC) to measure the performance of our model in the distinction between both categories. ROC curves were graphically represented. Additionally, the image processing performance of the network was determined by calculating the time required for the CNN to provide output for all images in the validation image dataset. The network’s classification was compared to the specialists’ analysis, the latter being considered the gold standard. Statistical analysis was performed using Sci-Kit learn v0.22.2 [12].

## 3. Results

### 3.1. Construction of the Network

**Figure 3:**
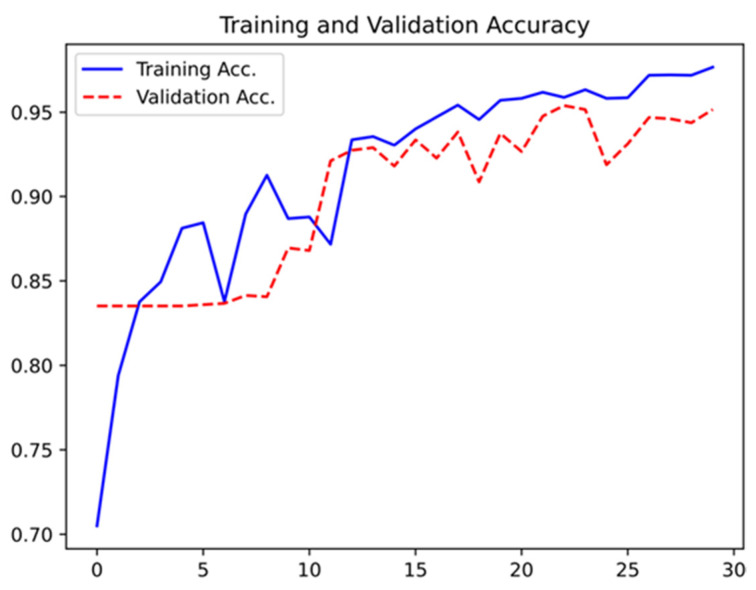
Evolution of the accuracy of the deep neural system during training and validation phases, as the training and validation datasets were repeatedly inputted in the neural network.

Seventy-two patients undergoing DAE between January 2020 and May 2021 were enrolled in this study. From these samples of patients, a total of 6740 frames were extracted (5345 showing normal mucosa and 1395 showing angioectasia). The validation dataset included 1348 images (20% of the extracted frames). It was composed of 279 (21%) images showing angioectasia and 1069 (79%) images with normal mucosa. The CNN evaluated each image and predicted a classification (normal mucosa vs. angioectasia, which was compared with the classification provided by the specialists. The network showed an increasing accuracy as data were repeatedly inputted into the multi-layer neural network (Figure 3).

### 3.2. Overall Performance of the Network

**Figure 4:**
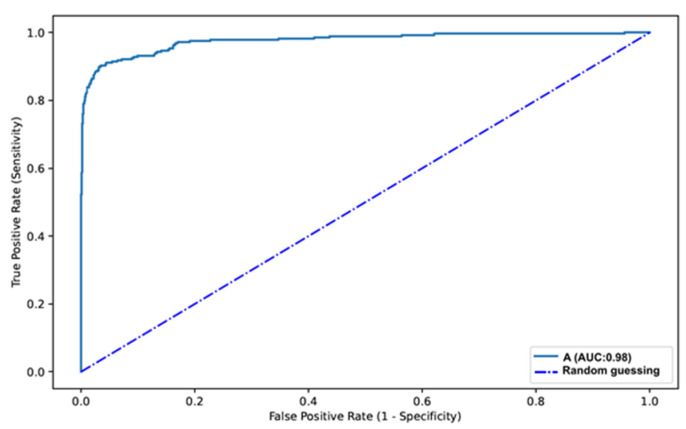
ROC analysis of the network’s performance in the detection of gastrointestinal angioectasia vs. normal mucosa. A—Angioectasia.

The distribution of results is displayed in Table 1. Overall, our automated system had a sensitivity of 88.5%, a specificity of 97.1%, a positive predictive value of 88.8% and a negative predictive value of 97.0%. Overall, the network had an accuracy of 95.3%. The AUC for predicting the presence of angioectasia was 0.98 (Figure 4).

### 3.3. Computational Performance of the CNN

The time required for providing classification for each image in the validation dataset was calculated. The CNN completed the reading of the validation image set in 9 s. This translates into an approximated reading rate of 6.4 ms/frame.

## 4. Discussion

In the last decade, we assisted the development of AI systems for application to several diagnostic modalities. Studies on the implementation of CNNs to several endoscopic modalities produced promising results [11,12,13,14,15]. In this retrospective study, we developed a pioneer deep learning algorithm for automatic detection of gastrointestinal angioectasia, with high sensitivity, specificity and overall accuracy.

Obscure gastrointestinal bleeding is the most common indication for DAE. Most often, patients presenting with OGIB, either overt or occult, undergo the previous investigation with capsule endoscopy (CE). The diagnostic yield of CE and DBE for the detection of small bowel angioectasia is similar (62% vs. 56%, *p* = 0.16) [5]. Additionally, the diagnostic yield of DBE significantly increases to 75% after a previously positive CE [5]. Nevertheless, rebleeding rates of patients undergoing therapeutic DAE are high, leading to the need for multiple treatment sessions, repeated use of red blood cell transfusions and hospital admissions [16,17,18]. Much of this high rate of rebleeding may be explained, in part, by the low yield of current diagnostic techniques, particularly CE and DAE, for the detection of small bowel vascular lesions, namely angioectasia [5]. Indeed, the rate of rebleeding after negative DAE is significant (38–40%) [19,20].

Recent advances in both computational power and high-definition endoscopy potentiated the development and application of AI systems in luminal endoscopy. In the last decade, several studies focused on the development of these tools for application to conventional esophagogastroduodenoscopy and colonoscopy. These works have mainly focused on the development of algorithms for the detection and characterization of premalignant or malignant lesions [21,22,23]. The first commercial AI solution for automatic identification of colorectal polyps received clearance by the U.S. Food and Drug Administration [24]. 

The development of AI mechanisms for automatic detection of gastrointestinal lesions mainly focused on CE for the detection of small bowel angiectasia, and to our knowledge, this is the first study to propose a CNN for use in DAE and for the detection of angiectasia in different segments of the gastrointestinal tract. Several studies focused on the computer-aided detection of small bowel vascular lesions in CE. Indeed, OGIB is the most common indication for both CE and DAE, and vascular lesions, particularly angioectasia, are the most common etiology. The group from Noya and colleagues was the first to describe the results of a study in which they developed an AI algorithm for automatic detection of small bowel angiectasia [25]. Their model had a sensitivity of 90%, a specificity of 97% and an AUROC of 0.93. In 2019, Leenhardt et al. reported a CNN capable of detecting angioectasia with a sensitivity of 100% and specificity of 96% [26]. These results were later replicated by Tsuboi et al., who developed a deep learning system with a high diagnostic yield for the detection of angioectasia, with a sensitivity of 98.8% and specificity of 98.4% [27]. 

Argon plasma coagulation through DAE is the most common form of treatment in patients presenting with OGIB who have angioectasia found during the investigation. Although DAE plays a more prominent role as a therapeutic rather than a first-line diagnostic method, its diagnostic yield for angioectasia is similar to CE, and treatment can only be applied if accurate identification of lesions is possible. The limitations in the diagnostic efficiency of both CE and DAE may explain the significative rate of rebleeding after negative DAE [20]. The application of deep learning algorithms to DAE for automatic detection of angioectasia is expected to significantly improve its yield in the diagnosis of these vascular lesions. Considering the dual role of DAE simultaneously as a diagnostic and therapeutic technique, a more efficient screening will probably contribute to more effective treatments, which may ultimately lead to better patient outcomes and lower rebleeding rates. The results from our study lay the foundations for further development of AI-based models for use in DAE.

This study has several highlights. First, to our knowledge, it is the first study to evaluate the performance of a CNN applied to DAE. Additionally, this is the first study describing the development of a deep learning system for the automatic detection of angioectasia applied to endoscopic modalities other than CE. Second, our algorithm demonstrated high performance levels. The current results are promising regarding the potential diagnostic benefits resulting from the application of these new algorithms. Third, our algorithm was built using images from two different enteroscope models, which may increase the applicability of our findings. Finally, our neural network had a high image processing capacity, with approximate reading rates of 155 frames per second.

Some limitations must be acknowledged. First, this study was of the retrospective design, including patients from a single center. The application of our system to clinical practice depends on the confirmation of our results in prospective studies, including patients from several centers in order to ensure an adequate dataset variability. Second, this proof-of-concept study was based on a small cohort, using a limited number of images. Therefore, no definite conclusions can be drawn regarding the clinical impact of this technology. Larger datasets, including data from a larger cohort of patients, are required to increase the robustness of our model. Third, this study focuses uniquely on the detection of gastrointestinal angiectasia and does not provide a classification of their bleeding potential, which may have a significant clinical impact. Subsequent studies on the development and application of AI algorithms to DAE must take this into account, as the identification of angioectasia with higher bleeding probability may significantly impact treatment and the risk of rebleeding. Finally, our model should be tested in real-time enteroscopy exams before definite conclusions regarding its real clinical significance can be withdrawn. These systems are only complementary to thorough endoscopic exploration as the software may also miss lesions that are beyond the reach of the endoscope lens or hidden between folds.

## 5. Conclusions

The influence of AI on endoscopic practice is expected to grow in the near future. This study evaluated the performance of a CNN-based model on the automatic detection of gastrointestinal angioectasia. We believe that the application of deep learning algorithms to advanced enteroscopy techniques may have a decisive impact in improving the care of patients with recurrent OGIB. The good performance of our model lays the foundations for future exploration of AI technologies in this subset of patients.

## Figures and Tables

**Table 1:** Distribution of results.

		Final Diagnosis	
		Angioectasia	Normal Mucosa
CNN	Angioectasia	247	31
	Normal mucosa	32	1038

## Data Availability

Data available upon reasonable request to the corresponding author.
